# HIV-related stressors and psychological functioning among adolescents living with perinatal HIV in KwaZulu-Natal, South Africa

**DOI:** 10.3389/fpubh.2025.1593387

**Published:** 2025-06-18

**Authors:** Thabani Nyoni, Wendy Auslander

**Affiliations:** ^1^School of Social Work, Faculty of Health, Dalhousie University, Halifax, NS, Canada; ^2^Brown School of Social Work, Washington University in St. Louis, St. Louis, MO, United States

**Keywords:** stigma, mental health, adolescents, perinatal HIV, South Africa

## Abstract

**Introduction:**

Adolescents with perinatally acquired HIV (APHs) in sub-Saharan Africa (SSA) face significant psychological challenges due to HIV-related stressors such as stigma and the burden of chronic illness, which can negatively impact mental health and antiretroviral therapy (ART) adherence. Mental health issues, including depression and anxiety, are common among APHs and often compounded by poverty, orphanhood, and stigma affecting both APHs and their caregivers. This study examines associations between HIV-related stressors (stigma experienced by APHs and their caregivers and the caregiver-perceived burden of the child’s illness) and psychological functioning (depressive symptoms, anxiety, and self-esteem) among APHs in South Africa.

**Methods:**

This cross-sectional study analyzed baseline data from the VUKA Family Program, an intervention in KwaZulu-Natal, South Africa (2014–2019). The sample included 315 APHs (aged 9–15) and their caregivers. Descriptive statistics assessed psychological functioning and HIV-related stressors. Multivariate regression analyses examined associations between APH-perceived stigma, caregiver-experienced stigma, caregiver-reported burden of the child’s illness, and psychological functioning outcomes.

**Results:**

Most APHs (*M* = 11.94 years, SD = 1.55, 52% female) reported high self-esteem (*M* = 86.3, SD = 10.1) despite experiencing elevated anxiety (*M* = 4.47, SD = 2.86) and depressive symptoms (*M* = 7.56, SD = 2.08). Caregivers (*M* = 40.48 years, SD = 12.96) experienced significant caregiving burden (*M* = 40.04, SD = 7.73). Both APHs (*M* = 19.6, SD = 3.80) and caregivers (*M* = 18.2, SD = 4.61) reported moderate levels of exposure to HIV-related stigma. Regression analyses showed that higher levels of APH-experienced stigma was associated to lower levels self-esteem (*b* = −0.75, *p* < 0.001), higher levels of anxiety (*b* = 0.29, *p* < 0.001), and depressive symptoms (*b* = 0.11, *p* < 0.001). Caregiver stigma predicted higher levels of APH anxiety (*b* = 0.11, *p* = 0.01) but was not significantly associated with self-esteem or depressive symptoms.

**Discussion:**

Findings highlight the critical role of HIV-related stigma in shaping APH mental health. Addressing stigma among APHs and caregivers through targeted interventions may improve psychological outcomes. Future research should explore self-esteem as a protective factor and validate depression scales for APHs in SSA.

## 1 Introduction

Mental health challenges among adolescents living with perinatal HIV (APHs) represent a significant yet overlooked public health concern in Sub-Saharan Africa (SSA). Following the World Health Organization (WHO) definition, adolescence is defined as the period from 10 to 19 years of age (1), and the current study uses this classification to guide inclusion and interpretation. Recent systematic reviews report that approximately 25% of adolescents living with HIV scored positive for psychiatric disorders, and 30–50% experienced significant emotional or behavioral difficulties (2, 3). These mental health issues often manifested as depression, anxiety, or behavioral disorders, which significantly compromised their quality of life and ability to manage their condition (4–6). Adverse mental health outcomes were closely linked to suboptimal antiretroviral medication adherence, a critical factor for achieving viral suppression and preventing disease progression (7, 8). Left untreated, psychological distress among APHs may further perpetuate HIV-related morbidity and mortality, undermining global efforts to achieve the UNAIDS 95–95-95 targets (9, 10). Addressing these challenges is therefore paramount for improving the health outcomes of APHs and ensuring the sustainability of HIV treatment programs in resource-constrained settings.

HIV-related stressors including disease-related stigma and social rejection experienced by APHs and their caregivers—are significant contributors to psychological distress (4, 5). Additionally, the burden of HIV illness on the family—manifested through caregiving demands, economic strain, and social isolation— further compounds psychological stress for both adolescents and their families (11). These stressors are exacerbated by broader structural inequities, including poverty and limited access to mental health care, leaving APHs especially vulnerable to suboptimal psychological functioning (2, 12). Although some studies have linked these stressors to adverse psychosocial functioning outcomes (13–15, 45), others have reported good psychological functioning among APHs, potentially attributable to protective factors like social support and family cohesion (8, 16, 17).

Although there is growing body of research that has assessed the psychosocial functioning of adolescents living with HIV in SSA, critical gaps remain. Much of the existing evidence, including studies by Cluver et al. (13), Nabunya and Namuwonge (18), and Adeyemo et al. (19), focuses broadly on adolescents living with HIV without distinguishing the unique experiences and vulnerabilities of APHs. Furthermore, many studies that investigated HIV-related stressors and mental health among APHs relied on qualitative methods, limiting generalizability (17, 20, 21). Evidence is also limited regarding the degree of APH’s exposure to risk factors and the extent to which these risk factors are associated with mental health problems. Few studies have quantified the levels of exposure to stressors such as stigma or caregiving burden or systematically examined their associations with psychological outcomes (46, 47). There is, therefore, an urgent need for research that not only describes the levels of exposure to HIV-related stressors but also explores their adverse impacts on psychosocial functioning among APHs.

Prior research in SSA highlights the multifaceted challenges faced by APHs, including the experience of living with a stigmatized chronic illness and residing in families affected by HIV/AIDS. These families are often marked by caregiving responsibilities or the loss of family members and income, compounded by impoverished conditions and limited access to care and support (16, 47). Such contextual factors intensify the psychological and emotional toll on APHs. Both qualitative and quantitative studies have found that greater exposure to HIV/AIDS-related stressors is associated with adverse psychological outcomes. For example, Betancourt et al. (17) and Hejoaka (11) describe how HIV-related stigma, and the burden of caregiving negatively impact the mental health of both children and their families. Similarly, Earnshaw et al. (46) identified a significant correlation between HIV stigma and increased risk of depression, while Bhana et al. (16) and Nestadt et al. (47) emphasize the compounded challenges of poverty, stigma, and family caregiving in resource-limited settings.

The present study is informed by Lazarus and Folkman’s (22) Transactional Model of Stress and Coping, which conceptualizes psychological stress as a process arising from an individual’s appraisal of environmental demands in relation to their perceived resources to manage them. Within this framework, stressors such as HIV-related stigma and family caregiving burden are seen not as inherently distressing, but as becoming so when perceived to overwhelm an adolescent’s coping capacity. The model also emphasizes the role of contextual and interpersonal factors—including family relationships and social support—as mediators or moderators of psychological outcomes. This theoretical orientation provides a useful lens for examining how exposure to HIV-related stressors may shape the mental health of APHs in resource-limited settings. Drawing on this framework and prior empirical evidence, the present study hypothesizes that APHs who experience higher levels of HIV-related stigma and greater family burden due to HIV will report significantly higher levels of depressive and anxiety symptoms, and lower self-esteem, compared to their peers with fewer such stressors. Accordingly, this study addresses two key research questions: (1) What are the levels of exposure to HIV-related stressors (i.e., HIV-related stigma experienced by APHs and their caregivers, and caregiver-reported burden of the child’s illness on the family)? (2) To what extent are these HIV-related stressors significantly associated with psychological functioning (i.e., depressive symptoms, anxiety, and self-esteem) among APHs?

## 2 Materials and methods

### 2.1 Study sample

This study utilized baseline data from the VUKA Family Program, a 12-session psychosocial family intervention. Conducted in KwaZulu-Natal, South Africa from 2014 to 2017. The VUKA program included 315 adolescents living with perinatal HIV (APHs) between the ages of 9 and 15 and their caregivers (12). Although the WHO defines adolescence as ages 10–19, participants aged 9 were included due to their early psychosocial vulnerability and developmental proximity to adolescence. The study name “VUKA,” a Zulu word meaning “wake up,” aimed to improve health, mental health, and behavioral outcomes for APHs. The KwaZulu-Natal province (KZN) has been considered the epicenter of the HIV epidemic in South Africa due to its high incidence and prevalence rates (23, 24). At the time of the study’s data collection, KZN had 11.4 million people living with HIV, constituting 19.7% of the provincial population and the second-highest number among South Africa’s nine provinces (23). By 2019, KZN had more people living with HIV (over two million) than Northwest, Limpopo, Western Cape, Free State, and Northern Cape combined (23).

The study was approved by the Human Subjects Institutional Review Board in both the United States and South Africa, as well as by the relevant hospitals and clinics involved in the research project, including R. K. Khan Hospital, Prince Mshiyeni Memorial Hospital, KwaNdengezi Clinic, and KwaMashu Clinic. Participants were recruited from four pediatric HIV outpatient treatment clinics, all of which were staffed by medical officers (general practitioners) with pediatric expertise or pediatricians, supported by nurses and lay counselors. Study participants were eligible for the study if they met the following criteria: (1) the adolescent was perinatally HIV-infected; (2) the adolescent was aware of their HIV-positive status; (3) the adolescent was on antiretroviral therapy (ART); (4) the adolescents and caregivers were Zulu or English speaking; (5) the APH’s healthcare provider was a staff member at one of the study clinics; and (6) each adolescent was accompanied by one caregiver for recruitment into the study. The final sample for the study included 315 APHs and 315 caregivers (age 18 + years) from the four HIV clinic sites.

### 2.2 Consent and data collection procedures

For the consent and assent procedures, caregivers were provided with comprehensive information about the study, including its purpose, procedures, and the time commitment required for participation. Researchers addressed all questions regarding participation, assuring caregivers that their decision to participate would not affect their position or treatment at their medical clinic or elsewhere. Caregiver consent was mandatory for adolescent participation. Once caregiver consent was obtained, the project research staff gave the child assent forms and explained the study. Both consent and assent forms were available in Zulu and English.

Following caregiver consent, researchers obtained written assent from the adolescent participants. The research staff explained the study to each adolescent using developmentally appropriate, plain-language descriptions of the study’s purpose, what participation involved, and their right to withdraw at any time without penalty. Verbal and written assent procedures were designed to ensure that adolescents understood what the study entailed and felt comfortable with their participation. The assent process emphasized voluntary involvement, confidentiality, and the limits to confidentiality in cases where safety concerns—such as suicidality or abuse—were disclosed. Adolescents were given ample time to ask questions before signing the assent form. These procedures were consistent with ethical guidelines outlined by the institutional research ethics board (IRB).

Caregiver consent forms detailed all aspects of the study, including the handling of data, the study’s purpose, location, activities, information to be provided, and the duration of assessments. The research team assured participants that confidentiality would be maintained unless there were concerns that required involving authorities, such as suicidality, homicidality, or child abuse. Participants were also informed that any disclosed, observed, or inferred child abuse must be reported to protective authorities. Adequate time was allowed for questions about the consent forms and the study. Original consent forms were securely stored in locked file cabinets at one study site, while copies were provided to caregivers and youth.

Following enrollment and consent, caregivers and children individually completed baseline interviews conducted separately by research team members. Each interview lasted approximately 1 h. Caregivers received $30 to compensate for their time and their child’s time spent with the interviewer.

### 2.3 Variables and measures

#### 2.3.1 Psychological functioning

This study assessed psychological functioning which included self-esteem, anxiety, and depressive symptoms.

##### 2.3.1.1 Anxiety (APH report)

Anxiety symptoms were measured using the APHs’ self-report responses to the 14-item State–Trait Anxiety Index-Child scale (STAI-C). This scale evaluated current manifestations of anxiety symptoms and a general tendency to be anxious (25). The STAI-C included items such as “I worry a lot of the time,” “It is hard for me to get to sleep at night,” and “I have bad dreams,” with responses of 0 (‘No’) to 1 (‘Yes’). The total scale score was created from individual items, with higher scores indicating higher levels of anxiety symptoms. The internal consistency reliability for this study’s STAI-C anxiety total score was Cronbach’s *α* = 0.77.

##### 2.3.1.2 Depressive symptoms (APH report)

Depressive symptoms were assessed using the APHs’ self-report responses to the 10-item Children’s Depression Inventory (CDI) short-form scale (26). Each item is rated based on the degree of depression symptomatology experienced in the last 2 weeks, using a set of three response choices for each item. For example, the item “sadness” includes the choices: 0 = “I am sad once in a while”; 1 = “I am sad many times”; and 2 = “I am sad all the time.” The total scale score, with higher scores indicating higher levels of depressive symptoms, was used. In the current study the Cronbach’s alpha coefficient was 0.48, and concurrent validity was demonstrated by correlating the depression total scale score with the anxiety scale (*r* = 0.41, *p* < 0.001).

##### 2.3.1.3 Self-esteem (APH report)

Self-esteem was measured using the APHs’ responses to the 20-item child version of the Tennessee Self-Concept Scale (TSCS-2) (27). The TSCS-2 assessed self-esteem components (identity, satisfaction, and behavior) across six domains (physical, moral, personal, family, social, and academic) with response options ranging from 1 (‘Always true’) to 5 (‘Always false’). Higher total scale scores indicated higher levels of self-esteem. In the current study the TSCS-2 total scale Cronbach’s alpha coefficient was 0.73.

#### 2.3.2 HIV-related stressors

This study assessed three HIV-related stressors: the burden of a child’s HIV illness on the family, adolescent HIV stigma, and caregiver stigma.

##### 2.3.2.1 The burden of a child’s HIV illness on the family (caregiver report)

The burden was measured using caregiver responses to 15 items from the 27-item Family Stress Scale (28), focusing on life events, induced and developmental transitions, community violence exposure, and daily family hassles. Each item had a five-point Likert response option ranging from 1 = “Strongly agree” to 4 = “Strongly disagree.” High scores indicated a higher burden. In the current study, the scale Cronbach’s alpha coefficient was 0.83.

##### 2.3.2.2 Adolescent stigma (APH report)

HIV-related stigma was measured using APH self-report responses to a 9-item HIV/AIDS Illness-Related Stigma Scale (29, 30). Items addressed external and internal stigma, with responses on a 4-point Likert scale ranging from 1 = “Strongly agree” to 4 = “Strongly disagree.” Higher scores indicated higher HIV-related stigma. In the current study the TSCS-2 total scale Cronbach’s alpha coefficient was 0.62.

##### 2.3.2.3 HIV/AIDS illness stigma (caregiver report)

Caregiver experiences of stigma related to the child’s HIV illness were measured using a 9-item scale similar to the one used for adolescents (29, 30). Responses ranged from 1 = “Strongly agree” to 4 = “Strongly disagree,” with higher scores indicating higher stigma. The scale’s internal consistency reliability coefficient for the present study was *α* = 0.76.

#### 2.3.3 Socio-demographic and family characteristics

##### 2.3.3.1 Adolescent characteristics

Age, gender, age at HIV diagnosis, and age at HIV disclosure were recorded. Age was used as a continuous variable, and gender was coded as 1 = male or 2 = female. Age at HIV diagnosis and age at disclosure were used as continuous variables in regression models.

##### 2.3.3.2 Family characteristics

Caregiver characteristics included age, gender, and HIV status. Caregiver age was recorded as a continuous variable, gender was coded as 1 = female or 0 = male, and HIV status was coded as 1 = HIV positive or 0 = HIV negative. Family living arrangements were also re-coded, including whether the adolescent lived with their biological mother, the number of rooms in the household, the number of adults and children sharing the household, and family deaths. Each of these variables was used in regression models as either dichotomous or continuous variables, as appropriate.

### 2.4 Data management and analysis

A preliminary analysis of the raw dataset (*N* = 315) revealed over 5% missing data in two dependent variables (depressive symptoms, anxiety) and four independent variables (APH stigma, caregiver stigma, burden of child illness). Further examination assessed the missing at random (MAR) assumption. Missing data were addressed using multiple imputation by chained equations (MICE), with models tailored to each variable’s distribution and measurement properties (31, 32).

Data analysis included descriptive statistics (means, medians, standard deviations for continuous variables and frequencies/percentages for categorical variables). Before conducting performing the multiple regression analysis, covariates were selected based on preliminary bivariate correlation analyses between demographic characteristics, HIV-related stressors and psychological functioning outcomes. Sociodemographic variables with statistically significant associations (*p* < 0.05) with at least one dependent variable were retained as control variables in the multivariable models (see Table 1). All bivariate analyses were conducted using the raw dataset. Finally, multiple regression analyses were performed on imputed data, with diagnostic tests ensuring model assumptions were met, including checks for multicollinearity, leverage, influence, and outliers (33).

**Table 1:** Bivariate correlations between independent and dependent variables (*N* = 315).

Variable	Anxiety symptoms	Depressive symptoms	Self-esteem
Socio-demographics			
APH age	−0.10	0.03	0.15*
APH age at HIV diagnosis	0.02	0.16*	−0.04
Adults in household	0.03	0.30	−0.12*
Rooms in household	0.04	−0.04	−0.04
Children in household	0.08	0.30	−0.05
APH gender	0.03	0.30	−0.02
Caregiver age	−0.02	−0.04	0.09
HIV-related stressors			
Adolescent stigma	0.38***	0.20***	−0.29***
Caregiver stigma	0.16**	−0.03	−0.14**
Burden of child illness	0.04	0.11	−0.14*

*p < 0.05, **p < 0.01. Correlation coefficients represent Pearson’s *r*. APH, Adolescents Perinatally HIV-infected. Sample sizes ranged from 283 to 315 due to missing data.

## 3 Results

### 3.1 Sample socio-demographic and family characteristics

Socio-demographic characteristics of APHs and their caregivers are summarized in Table 1. The average age of APHs was 11.94 years (SD = 1.55), with 52% (*n* = 164) identifying as female. The average age at HIV diagnosis was 3.83 years (SD = 3.44), while the average age at disclosure was 9.50 years (SD = 2.65). Regarding family characteristics, 57% (*n* = 179) of APHs lived with their biological mothers, while 47% (*n* = 165) identified their mother as their primary medication supporter. Additionally, 93% (*n* = 291) reported the loss of a caregiver or family member. Caregivers had a mean age of 40.48 years (SD = 12.96), with 90% (*n* = 285) identifying as female and 68% (*n* = 194) reporting living with HIV. Regarding family living arrangements, APHs shared their households with an average of 3.57 children (SD = 2.56) and 2.3 adults (SD = 1.89). The average number of rooms per household was 4.30 (SD = 2.34).

### 3.2 Levels of psychological functioning

Descriptive statistics on psychological functioning among APHs, measured by self-esteem, anxiety, and depressive symptoms, are presented in Table 2. The mean self-esteem score was 86.3 (SD = 10.1) on a scale ranging from 52 to 100. Based on Fitts and Warren (27), this falls within the “very high” self-esteem category (70–100). The mean anxiety score was 4.47 (SD = 2.86) on a scale ranging from 0 to 13. Although no clinical cut-offs exist for this measure (25, 34), findings suggest that participants in the current study reported anxiety levels comparable to those observed in youth with perinatally acquired HIV who experienced distress related to serostatus disclosure (34). The mean depressive symptom score for the sample was 7.56 (SD = 2.08), with an actual range of 3 to 15 out of a possible range of 0 to 20. According to a study by Allgaier et al. (35), APHs in the current study scored similarly to a sample of medically ill children who were diagnosed with a depressive disorder.

**Table 2:** Socio-demographics, psychological functioning and HIV-related stressors (*N* = 315).

Variable	*N*	Range	M/ %	SD/*n*
Age characteristics				
APH age	315	9–15	11.94	1.55
APH age at HIV diagnosis	308	0–12	3.83	3.44
APH age at HIV disclosure	312	1–14	9.51	2.65
Caregiver age	315	18–78	40.48	12.96
Living arrangements				
Children in household	315	0–21	3.57	2.56
Adults sharing household	315	0–10	2.30	1.89
Rooms in household	315	1–20	4.30	2.34
Family characteristics				
APH sex (Female)	315	–	52.06	164
Caregiver gender (Female)	315	–	90.48	285
Lives with biological mother (yes)	313		57.19	179
Caregiver HIV status (HIV+)	285	–	68.07	194
Mother as treatment supporter (yes)	310	–	46.77	165
Lost caregiver/family member (yes)	314	–	92.68	291
Psychological functioning				
Self-esteem	302	52–100	86.3	10.1
Anxiety	299	0–13	4.46	2.85
Depressive symptoms	274	3–15	7.55	2.08
HIV-related stressors				
Adolescent stigma	288	9–33	19.63	3.80
Caregiver stigma	303	9–33	18.22	4.61
Burden of child HIV illness on family	294	19–68	40.04	7.73

* Variations in sample sizes are due to missing data. M, Mean; SD, Standard Deviation; %, Percentage of group.

### 3.3 Levels of exposure to HIV-related stressors

Table 2 summarizes the descriptive statistics on exposure to HIV-related stressors among APHs and their caregivers, measured by self-reported HIV-related stigma and caregiver-reported burden of the child’s illness on the family. The mean HIV-related stigma score among APHs was 19.6 (SD = 3.80) on a scale of 9 to 33, indicating a moderate level of stigma. Caregivers self-reported similar levels of stigma with a mean total score of 18.2 (SD = 4.61), and an actual range of 9 to 33. Additionally, caregivers perceived a high burden of their child’s illness, with a mean burden score of 40.04 (SD = 7.73) with an actual range of 19 to 68, indicating a moderate level of stigma.

### 3.4 Bivariate correlations between dependent and independent variables

Table 2 represents bivariate correlations between indepedent variables (i.e., socio-demographic characteristics, HIV-related stressors) and dependent variables (i.e, psychological functioning outcomes) among APHs. Among the sociodemographic variables, APH age showed a significant positive correlation with self-esteem (*r* = 0.15, *p* < 0.05), and APH age at diagnosis was positively associated with depressive symptoms (*r* = 0.16, *p* < 0.05). Therefore, these two variables were selected due to their theoretical relevance and significant associations with psychological functioning outcomes. Other variables, including number of children or adults in the household, caregiver age and APH gender did not show consistent or significant associations with psychological functioning and were therefore excluded to avoid overadjustment and retain model parsimony.

### 3.5 Multiple regression models to predict APH’S psychological functioning

Table 3 present findings from three multiple regression models examining the associations between HIV-related stressors and psychological functioning among APHs, controlling for APH age and age at diagnosis. Each model assesses a different psychological functioning variable: Model 1 focuses on self-esteem, Model 2 on anxiety symptoms and Model 3 on depressive symptoms. The independent variables include three HIV-related stressors: APH experienced HIV-related stigma, caregiver experienced HIV-related stigma and caregiver reported burden of child’s illness.

**Table 3:** Multiple regression models predicting APHs’ psychological functioning (*N* = 315).

Independent variables	Model 1: Self-esteem symptoms	Model 2: Anxiety symptoms	Model 3: Depressive symptoms
	(B, 95% CI)	(B, 95% CI)	(B, 95% CI)
APH stigma	−0.75 (−1.04, −0.46) ***	0.29 (0.20, 0.37) ***	0.11 (0.05, 0.18) ***
Caregiver stigma	−0.20 (−0.48, 0.09)	0.11 (0.03, 0.18) *	0.01 (−0.05, 0.07)
Burden of child illness	−0.17 (−0.31, −0.03) *	−0.01 (−0.06, 0.03)	0.03 (−0.01, 0.07)
APH age^1^	0.76 (−0.05, 1.47) *	−0.09 (−0.28, 0.11)	0.05 (−0.11, 0.21)
APH age at HIV diagnosis^1^	−0.30 (−0.57, 0.07)	0.02 (−0.07, 0.11)	0.10 (−0.03, 0.17) *
Intercept	65.6 (58.6, 77.7) ***	−1.66 (−5.14, 1.83)	8.05 (5.3, 10.79) ***
Adjusted *R*^2^	0.08 ***	0.05 *	0.04 *

*p ≤ 0.05, **p ≤ 0.01, ***p ≤ 0.001.

1 Control variables.

In Model 1, higher levels HIV-related stigma among APHs was significantly associated with lower levels of self-esteem (*b* = −0.75, 95% CI [−1.04, −0.46], *p* < 0.001). Additionally, greater caregiver-reported burden of the child’s illness was significantly associated with lower levels of self-esteem (*b* = −0.17, 95% CI [−0.31, −0.03] *p* = 0.03). However, caregiver-experienced HIV-related stigma was not significantly associated with APH self-esteem. The model explained approximately 8% of the variance in self-esteem scores (Adjusted *r*^2^ = 0.08).

In Model 2, both adolescent-experienced and caregiver-experiences of HIV-related stigma were positively associated with higher levels of anxiety symptoms among APHs. More specifically, APHs reporting higher HIV-related stigma had significantly higher levels of anxiety (*b* = 0.29, 95% CI [0.20, 0.37], *p* < 0.001), and higher caregiver-experienced HIV-related stigma was also associated with higher levels of anxiety symptoms among APHs (*b* = 0.11, 95% CI [0.03, 0.18], *p* = 0.01). However, caregiver-reported burden of illness was not a significant predictor on anxiety symptoms in this model. The model accounted for about 5% of the variance in anxiety symptoms (Adjusted *r*^2^ = 0.05).

Model 3 showed that only APH-experienced HIV-related stigma was significantly associated with higher levels of depressive symptoms (*b* = 0.11, 95% CI [0.05, 0.18], *p* < 0.001). Neither caregiver-experienced stigma nor burden of the child’s illness was significantly associated with depressive symptoms. The model explained 4% of the variance in depressive symptom scores (Adjusted *r*^2^ = 0.04).

## 4 Discussion

### 4.1 Levels of psychological functioning among APHs

The current study found that APHs experience elevated levels of depressive symptoms and anxiety, aligning with prior quantitative research in SSA linking HIV-related stressors to psychological distress (3, 36). However, these rates were higher than those reported in similar studies in South Africa and Uganda (2, 8). This discrepancy may be attributed to unique contextual factors including HIV-related stigma, mental health stigma, and the burden of caregiving within extended and non-biological kinship networks—which may contribute to heightened psychological distress (2, 16, 17). These structural and familial dynamics likely intersect with patterns observed in the current study including high caregiver HIV prevalence, caregiver loss, and delayed disclosure of HIV status—to shape APHs’ adverse psychological outcomes.

Surprisingly, the present study found that APHs reported high self-esteem despite elevated depressive and anxiety symptoms, contradicting conventional coping models, which link psychological distress to lower self-esteem (15, 37, 38). One possible explanation is that psychosocial resources such as positive parenting, social support, and coping mechanisms help maintain self-esteem even amid mental health challenges. Prior research on adolescents living with HIV suggests that strong social connections and effective coping strategies foster self-worth and mitigate the negative impact of distress (6, 20).

Moreover, high levels of self-esteem in this population may not indicate the absence of distress but rather reflect resilience and adaptive mechanisms developed in response to adversity (6, 8, 16, 17). As Skovdal and Daniel (48) illustrate, HIV-affected children in SSA cultivate resilience through social support, community participation, and caregiving roles, maintaining self-esteem despite stigma, discrimination, and socioeconomic hardships. This indicates that APHs may leverage personal and social resources to navigate challenges and achieve positive psychosocial outcomes. Because these findings diverge from conventional psychological models that assume a negative correlation between distress and self-esteem, further research is needed to explore the role of coping and resilience among adolescents living with HIV in SSA.

### 4.2 Levels of HIV-related stressors among APHs and caregivers

Descriptive findings of the HIV-related stressors indicate that APHs and their caregivers experience moderate to high levels of HIV-related stigma. The findings are consistent with prior research, which has identified stigma as a pervasive challenge for adolescents living with HIV (39, 40). The moderate levels of caregiver stigma observed in this study are consistent with findings from prior studies (5).

Last, results indicated that caregivers perceive a significant burden related to their child’s HIV illness. This finding builds on prior qualitative research documenting the emotional, social, and logistical challenges faced by families managing a child’s chronic condition, including daily medication routines, stigma concealment, and health-related caregiving responsibilities (11). By providing quantitative evidence, this study extends the existing literature and highlights the potential need for systemic support to alleviate the burden on families.

### 4.3 HIV-related stressors and psychological functioning among APHs

The findings from the current study indicate that HIV-related stigma experienced by APHs is significantly associated suboptimal psychological functioning (i.e., high levels of depressive symptoms, and anxiety, and low levels of self-esteem). These results are consistent with findings from several prior studies that reported a significant association between adolescent exposure to HIV-related stigma and suboptimal psychological functioning (5, 8, 20, 40). Healthy cognitive and behavioral functioning has been closely linked to positive self-esteem, which can be adversely affected by a high level of HIV-related stigma (16). Woollet and others (8) found that the perception that one is experiencing HIV-related stigma triggers internalized fear of gossip or isolation and discrimination by family and community members, leading to adverse psychological functioning outcomes.

Additionally, the current study indicates that HIV-related stigma experienced by the caregiver due to a child’s HIV illness was significantly associated anxiety symptoms among APHs. This finding was consistent with findings from another study conducted in KwaZulu Natal, South Africa among 2,477 parent–child pairs which found that parents’ reports of HIV stigma were associated with greater anxiety among children (5). The study by (5), and the current study are the only studies to date in SSA that assessed caregiver’s experiences of HIV-related stigma due their relationship with a child living with HIV. More research is needed to deepen our understanding of HIV-related stigma and its impact on both the caregivers as well as the APHs.

### 4.4 Implications for practice

Findings from this study highlight the urgent need for interventions that support the psychological well-being of APHs by addressing both individual and systemic factors contributing to distress. Because of the high levels of depressive symptoms and anxiety observed, integrating mental health services within HIV care settings is essential (2, 8). In alignment with the World Health Organization’s recommendation for integrated mental health services in HIV care, developing and evaluating models that provide comprehensive mental health support to HIV-infected youth is critical (41). Results from this study also provide evidence of the adverse effects of HIV stigma on the mental health of both APHs and their caregivers, thus, suggesting the need for family-based stigma-reduction interventions (5, 40). Community-driven stigma reduction campaigns promoting HIV awareness and challenging misconceptions can also help dismantle stigma at both individual and societal levels, fostering supportive environments for APHs and caregivers (42). Finally, given that caregivers reported significant stigma and burden, providing mental health resources, peer support groups, and financial assistance for families affected by HIV could help mitigate stressors (43). Integrating caregiver-focused programs within HIV clinics could enhance both APH and caregiver well-being. By addressing these public health challenges through targeted, evidence-based interventions, policymakers and practitioners can work toward improving the mental health and overall well-being of APHs in SSA.

### 4.5 Implications for future research

Future research should examine the role of self-esteem as a potential buffer against psychological distress among APHs, particularly in the context of stigma and caregiving burden. Investigating the mechanisms through which self-esteem interacts with these stressors could inform targeted interventions. Additionally, studies should explore caregiver-child interactions to identify supportive communication strategies that enhance resilience and well-being. Given the link between caregiver stigma and APH anxiety, qualitative research could provide deeper insights into how caregivers navigate stigma while supporting their children. Longitudinal research is needed to assess the long-term psychological impact of HIV-related stigma and caregiving burden on APHs, determining whether early interventions lead to sustained mental health improvements. Furthermore, future studies should validate depression scales for APHs in SSA and explore alternative measures with stronger psychometric properties to ensure more reliable assessments of psychological distress in this population. By advancing these knowledge gaps, future research can contribute to evidence-based programs that improve the well-being of APHs in SSA.

### 4.6 Limitations

The findings of this study should be considered in light of several limitations. First, missing data were present for several key variables including depressive symptoms, anxiety, APH stigma, caregiver stigma, and caregiver burden of child illness. Although multiple imputation by chained equations (MICE) was used to address missingness (31, 32), the potential for bias remains. Second, the depressive symptoms scale demonstrated low internal consistency reliability. Future psychometric work is needed to culturally and developmentally adapt mental health measures for APHs in SSA. Third, caregiver-reported data on adolescents’ age at HIV diagnosis and age at disclosure may be subject to recall bias, particularly given the retrospective nature of these reports. While interviewers were trained to clarify timelines and cross-check details when possible, there remains the potential for some recall bias for those two variables. Fourth, the data used in this study were collected between 2014 and 2016. While this limits the temporal generalizability of the findings, more recent research continues to document high rates of psychological distress among adolescents living with HIV in SSA, suggesting that the issues highlighted in this study remain relevant (18, 44). Finally, the sample was drawn from healthcare clinics in KwaZulu-Natal, South Africa, which may limit the generalizability of findings to APHs in different geographic, socioeconomic, or cultural contexts. Clinic-based recruitment may also introduce selection bias, as adolescents engaged in care may differ from those not accessing services. Despite these limitations, this study advances understanding of the psychosocial vulnerabilities of APHs by highlighting the mental health burden associated with HIV-related stigma and caregiving demands. These findings contribute to the growing literature on adolescent mental health in resource-limited settings and may inform the design of targeted psychosocial interventions to improve APH well-being in SSA.

## 5 Conclusion

Our findings indicate that APHs in SSA experience elevated depressive and anxiety symptoms in addition to moderate-to-high levels of HIV-related stigma and caregiver burden. Moreover, stigma experienced by APHs was significantly associated with lower self-esteem, higher anxiety, and increased depressive symptoms, while caregiver-experienced stigma contributed to higher APH anxiety. These findings highlight the psychological vulnerabilities of APHs and the intergenerational impact of HIV-related stigma. Addressing stigma and caregiver burden through family-centered mental health interventions and integrated HIV care models may help improve APH’s psychological well-being. Community-driven stigma reduction initiatives and mental health resources for caregivers could further alleviate distress. Overall, these findings highlight the urgent need for targeted mental health interventions within HIV care settings to enhance psychosocial support for APHs and their families, which could ultimately contribute to improved retention in HIV care and progress toward UNAIDS 95–95-95 targets.

## Data Availability

The raw data supporting the conclusions of this article will be made available by the authors, without undue reservation.
